# Microcosmos explorers: foldscope workshop for science outreach in Mexican schools

**DOI:** 10.1093/biomethods/bpad035

**Published:** 2023-11-24

**Authors:** Samantha López Clinton

**Affiliations:** Department of Bioinformatics and Genetics, Swedish Museum of Natural History, Stockholm SE-10405, Sweden; Centre for Palaeogenetics, Stockholm SE-10691, Sweden; Department of Zoology, Stockholm University, Stockholm SE-10691, Sweden; Division of Biological and Health Sciences, Metropolitan Autonomous University, Mexico City 04960, Mexico

**Keywords:** Foldscope, science outreach, microscopy, frugal science

## Abstract

Foldscopes are ultra-low-cost paper microscopes invented by Manu Prakash and Jim Cybulski at Stanford University. They are about as light as a pencil and waterproof, all whilst offering similar optic quality to traditional microscopes. Foldscopes do not require electricity or glass slides to be used, which increases the possibilities of their use in education and outreach activities with children or people with disabilities. In 2019, thanks to a material grant of 100 foldscopes from One World Science and additional purchased foldscopes, I designed and implemented a science workshop called *Exploradores del Microcosmos*, or Explorers of Microcosmos in English. The aim of the workshop was to help make microscopy more accessible, in particular at underfunded schools, and stimulate active learning about ecosystems and evolution in the participants. Within this article, I describe the workshop and relay my personal insights and reflections on its execution across multiple schools and groups in Mexico.

## Introduction

**Figure 1.**
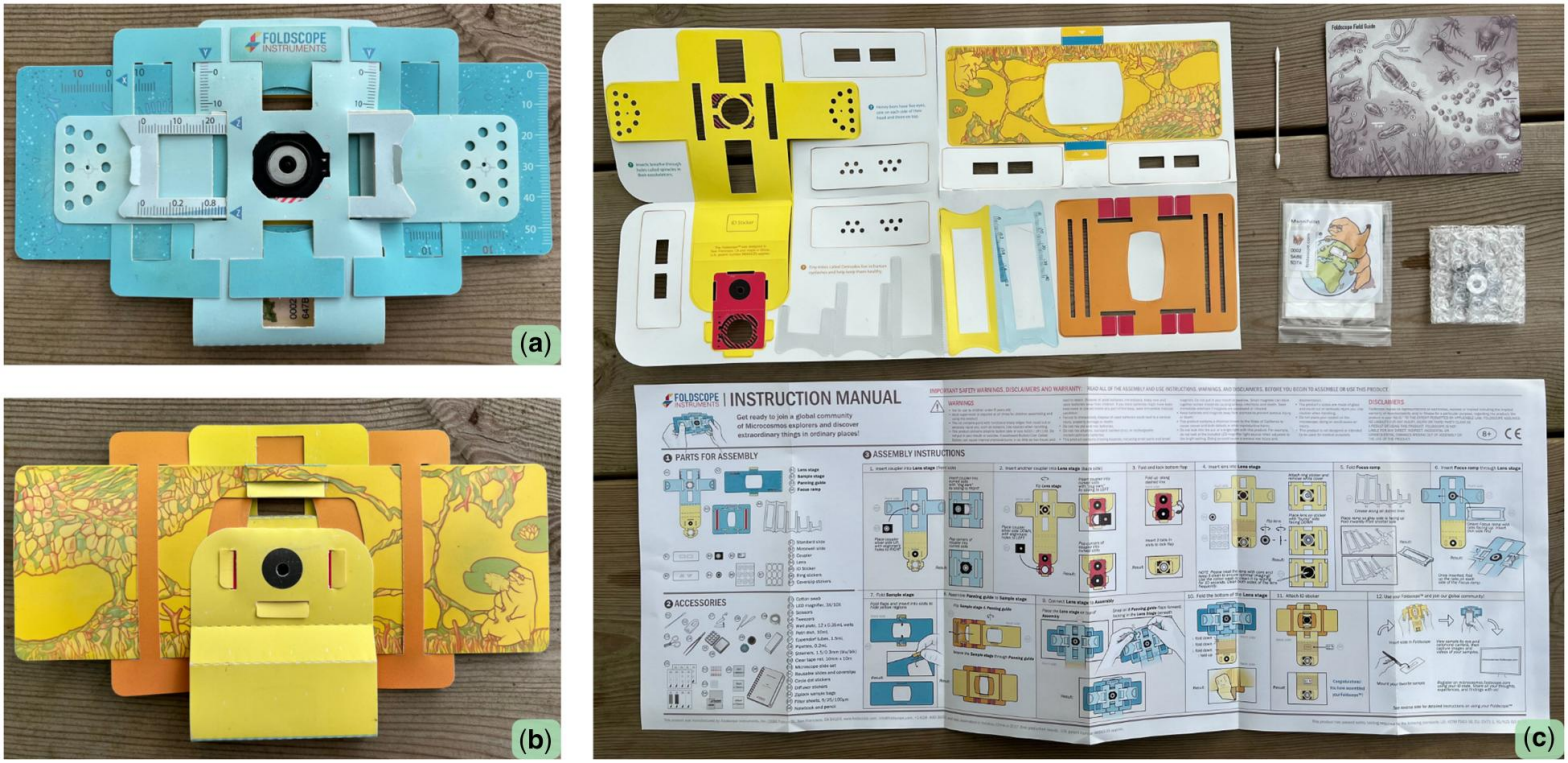
Foldscope photographs. Anterior (a) and posterior (b) sides of an assembled Foldscope. c) Not yet assembled Foldscope, a q-tip for lens cleaning, a foldscope field manual, a plastic bag with stickers, the lens and adapters, and the instruction manual - all included in each Foldscope.

Frugal science, a visionary concept of providing affordable and readily accessible scientific instruments, holds particular significance for regions and populations with limited resources [1, 2]. Amongst the pioneering innovations in this domain are foldscopes (Fig. 1), ultra-low-cost microscopes developed by Manu Prakash and Jim Cybulski at Stanford University [3]. Composed of waterproof paper, users assemble them in an origami style, offering optical quality and magnification akin to conventional microscopes. Foldscopes have improved scientific accessibility, emerged as a cornerstone in frugal science, and are now used in diagnostics, health, biodiversity science, and other fields [4].

A single foldscope costs around €8, and they are less expensive when purchased in bulk [5]. For example, a basic classroom kit of 20 of these costs around €33.3, a kit of 100 foldscopes has a cost of around €332 (around €3.3 per foldscope). Additional foldscope accessories are also available for purchase on their website.

Several characteristics distinguish foldscopes from traditional optical microscopes. Here, I describe the three most relevant ones for the Explorers of the Microcosmos workshop. The first is portability. Traditional optical microscopes are typically heavy and require stable, dedicated workspaces due to their delicate and complex components. Foldscopes, on the other hand, are lightweight and durable, such that they can be carried far and wide [3]. Secondly, foldscopes also eliminate the dependence of microscopy on electricity, as they can use external light sources to illuminate the image seen [3]. This is transformative for on-the-go scientific exploration and microscopy use in underserved regions. And thirdly, foldscopes incorporate a DIY (Do It Yourself) component that empowers users to assemble their own microscopes. This is paramount in education as it fosters a sense of ownership and curiosity, all whilst encouraging users to become familiar with the inner workings of this scientific instrument [4, 6].

These features make foldscopes a transformative tool in frugal science with the potential to democratize access to scientific exploration, and a powerful instrument in science education, particularly for resource-limited regions. As such, they have been used repeatedly in outreach and education initiatives [2, 6–11]. For a comprehensive review of both research and education projects carried out with foldscopes, see Ganesan *et al.* [4].

## Background to Explorers of the Microcosmos

Foldscopes are an excellent tool to develop outreach science activities. Here, I describe the use of foldscopes for educational purposes in the context of a workshop that I designed and taught called ‘Explorers of the Microcosmos’. This workshop was a way for me to complete a Social Service project to obtain my degree at the Metropolitan Autonomous University Xochimilco Campus (UAM-X, for its abbreviation in Spanish). In this section, I describe the development and aims of the workshop.

In most state universities in Mexico, a Social Service of at least 6 months is expected from every BSc student before attaining their degree. Social Service mainly consists of activities carried out by the student in benefit of society and the state. As my Social Service requirement, I secured a grant that allowed me to design and offer a science workshop in Mexico using foldscopes, a project which was approved by Dr. Marisa Arienti Villegas, at the Division of Biological and Health Sciences. In 2019, I received a material grant of 100 foldscopes from The People’s Microscope, an initiative from Field Projects International (FPI). After the success of the initial workshop phase, which will be referred to as ‘stage 1’, I acquired an additional 200 foldscopes through personal funding. This enabled the continuation of the workshop, now offered in exchange for a fee to cover expenses, which will be referred to as ‘stage 2’. In this article, I share the structure of the workshop together with my personal thoughts and reflections about how the workshop was carried out with the hope that it may offer valuable insights to those embarking on similar ventures.

The workshop aimed to make science and the discovery of the microcosmos more accessible to children and young adults, particularly in schools that did not have well-equipped biology laboratories. In this context, microcosmos is understood as the microscopic world (abiotic or biotic). It also aimed to leverage innate curiosity to generate questions—and knowledge—about the microscopic world that surrounds us and the role that it plays in ecosystems such as rhizospheres, bodies of water, and forests, as well as an introduction to the concept of evolution.

The workshop was targeted towards school children—ages 10–18, in a school setting. Like another microscope assembly workshop [12], Explorers of the Microcosmos was also open to any educators at the school who wanted to take part, with the goal of having them include the foldscopes in any pedagogical activity at the school at a later time. A few of the groups also included mixed classrooms, where some of the students had some sort of disability. Stage 2 foldscopes were used to offer the same workshop in various settings—such as universities, children groups, families, and research centres—and age groups ranging from around 8 to 70 years old.

Before implementing a workshop at a school, the institution was contacted and told about the project. The school was informed that the workshop was approved by the University, that it involved no cost to the school (except for stage 2 workshops), and that it was open to all students and members of staff. If the school was interested, at this stage they communicated their logistic possibilities such as projector availability, group size, or possible workshop locations within the school, such that I could adapt the material to available space and resources. Due to the social media presence of the workshop, I was also contacted by a few groups of people or other schools/universities to implement the workshop with their group. In total, the workshop was offered to 16 groups (Table 1).

**Table 1.** Locations and institutes at which the workshop was offered in 2019.

Type	Institute	City	Number of groups
School	*Instituto Flemming* (Flemming Institute)	Mexico City	2
	*ALAS Aprendizaje en Libertad* (ALAS Learning in Freedom)	Guadalajara	1
	*Centro Educativo Muralistas Mexicanos* (Education Centre Mexican Muralists)	Guadalajara	1
	*SIGNOS Secundaria y Bachillerato A.C.* (SIGNOS Secondary and High School A.C.)	Guadalajara	1
University	*Universidad Autónoma Metropolitana—Unidad Xochimilco* (Metropolitan Autonomous University—Xochimilco Unit)	Mexico City	2
University	*Centro Universitario de Ciencias Exactas e Ingenierías—Universidad de Guadalajara* (University Centre for Exact Sciences and Engineering, University of Guadalajara	Guadalajara	1
Research Centre	*Laboratorio de Futuros en Bioenergía, Centro de Investigación y de Estudios Avanzados del Instituto Politécnico Nacional—CINVESTAV* (Futures in Bioenergy Laboratory, Centre for Research and Advanced Studies of the National Polytechnic Institute—CINVESTAV)	Guadalajara	1
Other	*Comunidad Ecológica Los Guayabos* (Ecological Community ‘*Los Guayabos*’)	Guadalajara	1
	Various small scale workshops requested by private groups or advertised on social media (attendees ranged from around 8 to 70 years of age)	Guadalajara and Mexico City	6

## Structure

The science workshop was structured into seven distinct sections, each with particular objectives. In the following segment, I provide a description of each section whilst covering the underlying motivations that guided their development. A recapitulation of all workshop sections, in English and Spanish, is found in Supplementary Data 1 and 2.

Section ‘Questionnaires’ of the Explorers of the Microcosmos workshop consisted of an initial anonymous questionnaire (Table 2; refer to Supplementary Data 3 for a version in Spanish) distributed as printed copies to the students. Using the conditions shown in Table 3, the answers to questions 1, 2, and 4 were classified as correct and incorrect. Answers to question 5 were classified into three groups: organisms, parts of organisms, and others. Both classifications were recorded for comparison to those obtained from the final questionnaire.

**Table 2.** Initial questionnaire—Explorers of the Microcosmos.

	Date: Institution: Group: Age:
**1**	What is a **microorganism**?
**2**	What are **microscopes** and what are they for?
**3**	Have you ever observed anything under a microscope or a magnifying glass? If yes, what did you observe?
**4**	Do you know what an **ecosystem** is? And if so, why are they important? Can you name some ecosystem components?
**5**	In the microscopic world we can observe many different organisms that are too small to observe with the naked eye. Can you name a few of these organisms?

**Table 3.** Criteria used to classify answers to questions 1, 2, and 4 as ‘correct’.

Concept	Criteria to be considered ‘correct’
Microorganism	Any answer that mentions life (organisms, beings) at a small scale. Keywords: ‘impossible to be seen with the naked eye’, ‘microscopic’, ‘tiny’, ‘small’
Microscope	Any answer that mentions tools or equipment to see objects up close
Ecosystem	Any answer that mentions relationships or dynamics between biotic and abiotic factors

The questionnaire portions of the workshop were designed to probe the students’ existing knowledge of microscopy, microorganisms, and ecosystems. The primary objective was to assess the student’s baseline understanding and later compare their responses to the same questions upon completing the workshop.

Section ‘Guess the object’ engaged students in an intriguing challenge. During this activity, students were presented with a captivating array of images on a projector, each featuring everyday objects as observed under various microscope types. The collection of images encompassed items such as threaded needle, toilet paper, a used toothbrush bristle, zooplankton, a hair follicle, mites, butterfly wing scales, and a flower petal. Students would then be encouraged to use their powers of observation and deduction to identify the objects. Beyond the elements of fun and curiosity, the primary aim of this activity was to immerse the students in the microscopic realm. More specifically, it shows that there are various types of microscopes, for example, stereo, electron, or compound microscopes. The purpose of this exercise was to introduce the students to the many uses of different microscopes.

During section ‘What are microorganisms and why are they important?’, the students were organized into small groups of 2–5 members. Each group received a unique set of colour printed laminated illustrations sourced from the children’s book *Los mundos invisibles de los animales microscópicos* [13] or ‘The invisible world of microscopic animals’, in English. The book is also available in English as ‘Unseen Worlds: Real-Life Microscopic Creatures Hiding All Around Us’, published by What on Earth Books in 2019; in French as ‘Les Mondes Invisibles Des Animaux Microscopiques’, published by Actes Sud in 2016; and in Chinese as ‘Microworld (The Little Lives Like the Mysteries)’, published by Beijing United Publishing co., LTD in 2019.

**Figure 2.**
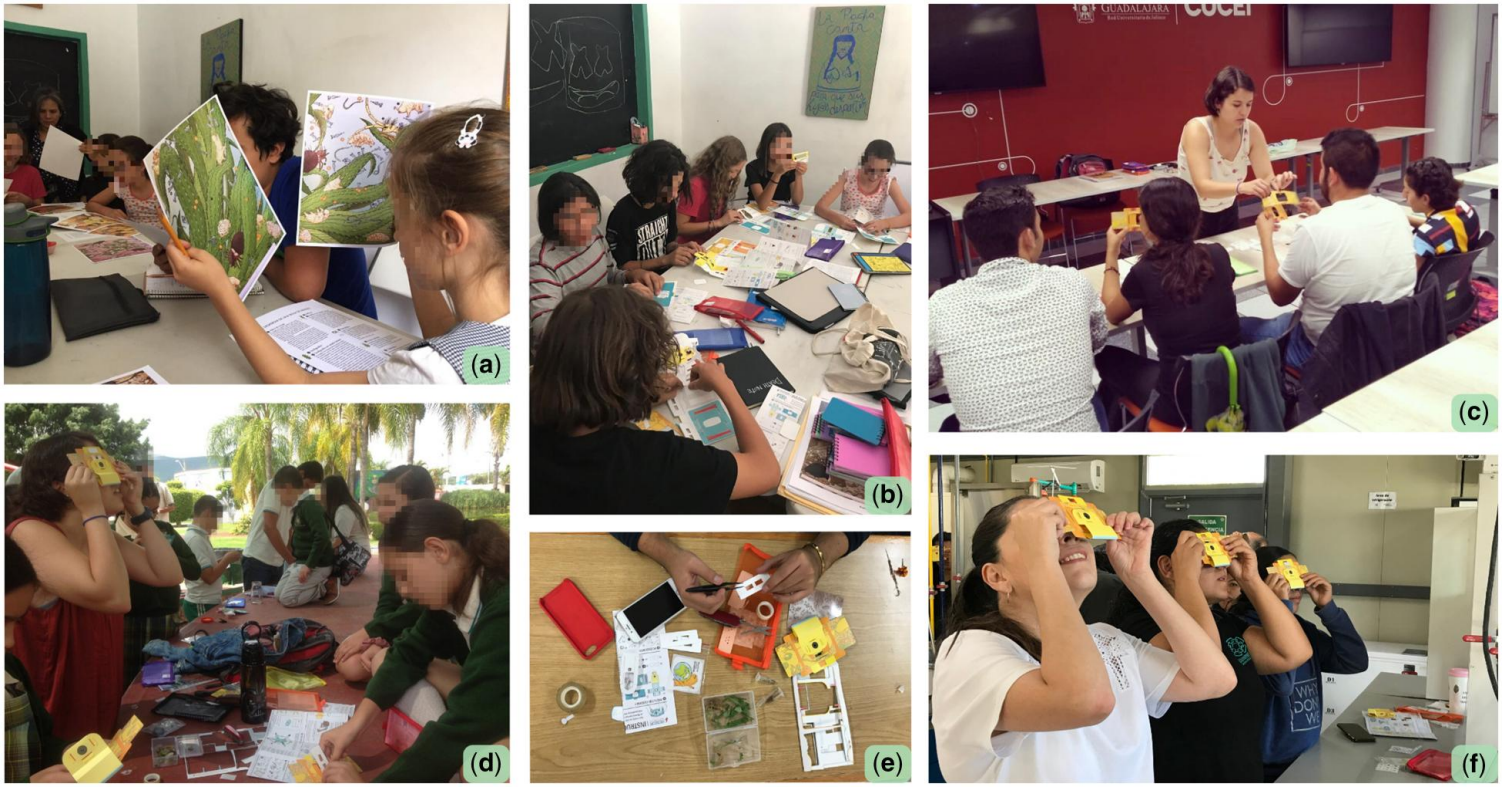
Collection of images from some of the activities in the workshop. a) Student observing the print-outs during section III; b) and c) Foldscope assembly in two different groups, section IV; d) Learning to load slides and visualise them in the Foldscope, section V. e) and f) Students preparing their own samples on the Foldscope paper slides, section VI.

The images vividly portrayed various microscopic ecosystems, each complemented by a concise narrative and a description of the organisms inhabiting the depicted environment (Fig. 2a). The ecosystems spanned a diverse range, encompassing settings such as a droplet of ocean water, a kitchen floor, a bed, a forest floor, and more. Students were encouraged to closely observe their assigned laminated images, choosing an organism that piqued their curiosity. Once the selections were completed, they took on the role of presenters for the whole class, elaborating on their chosen organism’s ecological role and describing why they were interested in this particular organism. This section’s main objective was to introduce the concept of ecology in a captivating and interactive manner, emphasizing the interconnectedness of all elements within an ecosystem, even if they are not visible to the naked eye. It was also important to introduce them the concept of evolution, and how the intricate relationships between the ecosystem components are the result of a long evolutionary history. A short script containing the broad definitions used during the course for key concepts (ecosystem, microorganism, evolution) is found in the Supplementary Data 4.

Section ‘Build your own foldscope’ consisted of the assembly of the foldscopes. Each student was given a foldscope, which came unassembled. For success of the workshop and to foment the independent use of the foldscopes, it was important that the students assembled their own and acquired a sense of ownership, curiosity, and care for their new microscope (Fig. 2b and c). As the foldscopes came with assembly instructions in English, and considering that the workshop was conducted in Mexico, where not all students may be proficient in English, we collectively assembled them step by step.

During section ‘Visualizing prepared samples’, students learned to place slides into their foldscope and how to manoeuvre the microscope to view the sample correctly (Fig. 2d). They did this using a set of fixed glass slides provided by foldscope in their ‘teacher kits’ (Supplementary Video 1). Slides included artery sections, rhizome, muscle, plant tissue, bone, and more. The goal of this section was to captivatingly teach the students to load slides onto their foldscopes, and adjust the settings to visualize the image properly.

In section ‘Exploration’, participants engaged in a hands-on exploration of their own. Armed with an assortment of organic materials at their disposal, including flowers, leaves, mosquito larvae, pond water, and insects, students undertook the task of crafting their own paper slides (Fig. 2e and f). Within the ‘teachers kit’ included in each foldscope set of 20 microscopes (but also available for purchase as the ‘assembled individual kit’, around €16), are droppers, scissors, and tweezers, all of which were made available to the students to facilitate this task. Additionally, rolls of clear tape were also provided for students to use as slipcovers in case they did not want to use the limited ones included with their foldscope. Encouraging independent thought and initiative, students were prompted to select and prepare their own samples for microscopic observation. The exploratory spirit extended to the immediate environment, where students could exercise their discerning eye in identifying samples suitable for slide preparation. This workshop section not only imparted practical skills in slide preparation but also nurtured creativity and resourcefulness, instilling the concept that the microscopic world is not confined to the laboratory, but is omnipresent and ready to be discovered and explored.

In section ‘Microcosmos network’, students were introduced to the online foldscope social network ‘Microcosmos’ (https://microcosmos.foldscope.com/), where people from all over the world can post their discoveries, ask questions, and connect with other explorers. The aim of this introduction was to allow the participants, and their teachers, to connect with other foldscope users across the globe. It is also a great opportunity to get ideas for more observations, or for foldscope ‘hacks’ to better visualize a sample.

Section VIII, "Final questionnaire", included the same questions as the initial one and was also anonymous, however, it included an additional set of feedback-related questions and about how participants would like to use it in the future (Supplementary Data 5). After each workshop, I provided all participants with my contact details (phone number and email) should they have any questions about their foldscope or observations they wanted to share.

## Workshop outcomes and personal insights

The following section contains personal reflections on which elements of the workshop were successful and which could be improved. Continuity proved to be an important factor for the workshop in general. A number of students reached out to me personally with their discoveries days after the workshop, given that I had provided my contact details. One group’s science teacher sent me pictures a few months after the workshop showing how the foldscopes were being used for a biology lesson. A collection of pictures obtained from the observations during the workshops is available in Fig. 2. Whilst granting my contact details was a personal decision, and should not be expected from most education providers, it is clear that it provided a fast and easy way for students and teachers to communicate their findings and ask questions after the workshop had long ended. Explorers of the Microcosmos would have likely benefited from having continuity in the form of a monthly, bimonthly or biannual visit to the school to discuss their findings and answer questions. This would also have been an ideal opportunity to collect answers to the questionnaires once again, in order to evaluate in what proportion the information was retained.

### Questionnaires

Due to time constraints, the questionnaire was not integrated into the stage 2 workshops. Additionally, in one instance, at the Education Centre Mexican Muralists, the questionnaires were omitted at the request of the teacher. This group consisted of 30 participants, comprising both children with and without disabilities. The teacher anticipated that the foldscope building section of the workshop might require additional time, which influenced the decision to prioritize other workshop components in this particular setting. In total, 59 students from the remaining institutions (Flemming Institute, ALAS Learning in Freedom and SIGNOS Secondary and High School) completed both questionnaires.

Comparison of questions 1, 2, and 4 of the initial and final questionnaires showed an increase in the number of correct answers by the students after completing the workshop (Table 4). Despite the low sample number, it appears that the content of Explorers of the Microcosmos enabled students to retain some of the concepts covered in the workshop. Another increase was apparent in the number of microorganisms (or parts of them) that the student could name (Supplementary Data 6).

**Table 4.** Correct answers recorded in initial and final questionnaires during the workshop.

Questions		Correct answers (from a total of 59)	
		Initial questionnaire	Final questionnaire
1	What is a **microorganism**?	34	41
2	What are **microscopes** and what are they for?	48	52
4	Do you know what an **ecosystem** is? And if so, why are they important? Can you name some ecosystem components?	6	29

Questionnaires, whilst being a valuable tool for assessment, can sometimes pose challenges in terms of student engagement. It was evident that a subset of participants began to exhibit signs of waning interest during the questionnaire phases, with some opting to divert their attention elsewhere. This underscores the need to find a balance between collecting essential data for accurate workshop evaluation and maintaining participants’ sustained engagement. Thus, a strategic adjustment in the workshop sequence may be warranted. Starting with section ‘Guess the object’, rather than the questionnaire, could potentially offer a more captivating and participatory introduction to the workshop, thereby reducing the risk of early disengagement amongst participants.

### ‘Guess the object’

This activity proved to be highly engaging for the majority of students, with particular enthusiasm stemming from the images of everyday items such as toothbrush bristles, toilet paper, and dental floss, which sparked lively discussions within the group. Overall, it seemed that this approach served as an excellent introduction to the field of microscopy.

### ‘What are microorganisms and why are they important?’

The material used in this exercise also elicited enthusiastic participation from the students especially when it came to the younger participants. Laminating the instructional material not only ensured its durability and reusability across diverse age groups but also allowed the students to closely examine intricate details within the images. The process of selecting their favourite organism and presenting it to the group seemed to foster familiarity with various microorganisms and underscore the vital roles each living entity plays within its ecosystem. This section presented an opportunity to delve into the role of evolution on the intricate relationships between ecosystem components.

### Build your own foldscope

Each student successfully assembled their own foldscope, with some receiving guidance from the workshop instructor, or even other students, when needed. This stage was deemed fundamental in the workshop’s inception. The reason for this is that the process of building their own learning tool can empower participants to demystify the instrument and take ownership of it and their discoveries, thereby making microscopy more accessible [6, 4].

### Visualizing prepared samples

This section, which involved students loading and visualizing fixed glass slides using their foldscopes, received positive feedback from the participants. The diversity in the samples proved to be particularly engaging. It was also evident that students found it easier to learn to manipulate the focus ramp and slide with glass slides, perhaps because the image tends to be sharper. It is worth noting that even the youngest workshop attendees successfully and safely utilized glass slides under adult supervision, without any accidents.

### Exploration

**Figure 3.**
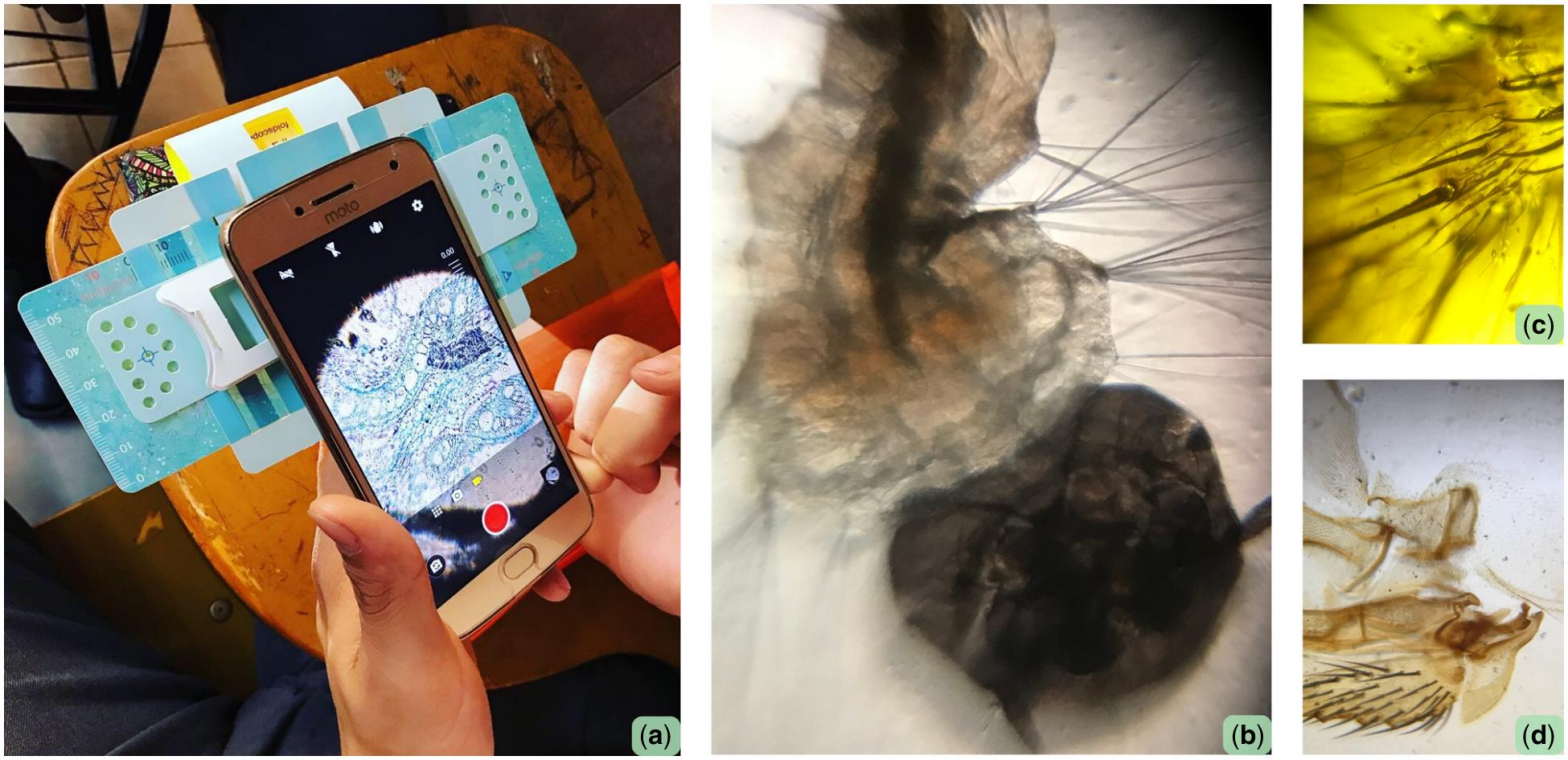
Images of different slides observed by the students during the workshops. a) a rhizome; b) a mosquito larva; c) a section of a spider leg; d) a section of a fly wing.

This section of the workshop allowed students to think outside of the provided samples and collect objects from their environment—be that in the classroom or outside of it (Fig. 3). It was perhaps, aside from the foldscope assembly, the most hands-on and captivating portion of the workshop. Hands-on learning, in this case through the use of foldscopes, is found to be more beneficial than just passively receiving information [11, 14, 15]. Tools such as the foldscope, which can be carried around and used independently, can aid students in performing open-ended experiments, which foment ‘science-specific skills’ [2, 6]. The students often needed help loading their samples onto the sticky side of the tape, or had trouble adjusting the lens to get a clear picture. However, once a few of the students had solved these issues themselves, they often aided the others, which enriched the discovery section of the workshop.

### Microcosmos network

Whilst it remained crucial for students to grasp the concept of the global microcosmos social network, and its role in sharing discoveries and gaining inspiration for their observations, this workshop segment likely warrants a more didactic approach than the one applied here. A potential solution could involve students actively creating and sharing their post(s) during the exploration section, as opposed to passively observing a screen with the website. It is also important to acknowledge that not all students have access to smartphones with cameras and that given the fact that the network is public and global, underaged participants would likely require parental approval before joining the platform. To circumvent this issue, the class could also work on creating their own posts after the workshop, during their science or biology lesson. This way, should the students be too young or lack a smartphone, the teacher could post the content for them.

## Conclusions

Explorers of the Microcosmos was a small-scale but successful science outreach workshop in Mexico which made use of the ultra-low-cost paper microscope—foldscope—invented by Manu Prakash and Jim Cybulski at Stanford University. The workshop was designed to captivate students, engage them in active learning, and channel their creativity through hands-on experiences in constructing their own foldscopes and observing various preserved and freshly collected samples.

Initial and final questionnaires conducted before and after the workshop revealed an improvement in the students’ comprehension of microorganisms, microscopes, and ecosystems. Larger sample sizes, a control, and more rigorous evaluations would be needed to establish whether this improvement was attributable to workshop participation. The foldscope remains one of the most momentous inventions in the context of frugal science and science democratisation, and as such, a key educational tool for underprivileged schools and regions.

Continued sharing of personal experiences and insights from similar initiatives is imperative and an essential resource for educators worldwide embarking on comparable endeavours.

## Supplementary Material

bpad035_Supplementary_Data

## Data Availability

Questionnaires and workshop structure are available in the main article and the supplementary. Responses to the questionnaires are available upon request.
