# Supplementary material for: Microcosmos explorers: foldscope workshop for science outreach in Mexican schools
Source: Biol Methods Protoc. 2023 Nov 24;8(1):bpad035. doi: 10.1093/biomethods/bpad035 (PMC10759293; doi:10.1093/biomethods/bpad035)
Supplement: bpad035_Supplementary_Data [file bpad035_supplementary_data.zip › BMP-2023-067_REVISED_Supplementary_materials.docx]

Supplementary 1.

| Supplementary Table 1. English. Explorers of the Microcosmos workshop sections, activities and aims. | |
| --- | --- |
| Section and activities | Aims |
| 1. **Initial questionnaire**   Students wrote individual and anonymous answers on a paper print-out with a few questions about microscopy, microorganisms and ecosystems. | Evaluate students previous knowledge |
| 1. **“Guess the object”**   Students were presented with several mystery microscopic images (dental floss, toilet paper, mites, butterfly scales, zooplankton, hair follicle…) and had to guess what they were. | Engage the students and introduce the microscopic universe |
| 1. **“What are microorganisms and why are they important?”**   Small groups of students (2 to 5 members) were given a set of laminated images of different ecosystems from XXXX. Students were instructed to choose a microorganism and explain to the rest why they thought it was interesting. | Help the students realise that microorganisms play a vital role in many ecosystems |
| 1. **Build your own foldscope**   Students were given their own foldscope, which comes unassembled, and instructed to assemble them step-by-step while the instructor physically showed the procedure. | Show the students how to build their own microscope, thereby also acquiring a sense of ownership, curiosity and care for their instrument |
| 1. **Visualising prepared samples**   Students learned to use the foldscope with a set of fixed slides included in the “teacher foldscope” (rhizomes, muscle, arteries, bone, foliar tissue, pollen and bacteria) provided by Foldscope Instruments. | Teach the students to insert and visualise slides with their foldscope using captivating samples |
| 1. **Exploration**   Students were instructed to prepare their own paper slides included in the foldscope. Samples could stem from facilitator-provided material (flowers, leaves, insects, pond/puddle water, mosquito larvae…) or found in the workshop location. | Teach the students to prepare their own slides and use their creativity to select objects from their surroundings to observe |
| 1. **Microcosmos network**   Students were introduced to the social foldscope network “Microcosmos” and shown how they share their own discoveries. | Foment the use of the foldscope outside the workshop and even outside the classroom. |
| 1. **Final questionnaire**   Students answered another questionnaire containing the same questions as the initial one, in addition to a few more about their thoughts on the workshop and their future use of the foldscope. | Compare responses between initial and final questionnaires and evaluate the success of the workshop. Incorporate feedback in the next workshop if possible. |

Supplementary 2.

| Supplementary Table 2. Spanish. Secciones, actividades y objetivos del taller Exploradores del Microcosmos. | |
| --- | --- |
| Sección y actividades | Objectivos |
| 1. **Cuestionario inicial**   Les estudiantes contestaron de manera individual y anónima sus respuestas a un cuestionario impreso con preguntas sobre microscopía, microorganismos y ecosistemas (Supplemento XX) | Evaluar el conocimiento previo de los estudiantes |
| 1. **“Adivina el objeto”**   Les estudiantes fueron presentados con varias imágenes microscópicas (hilo dental, papel de baño, ácaros, escamas de mariposa, zooplankton y un folículo de cabello) | Involucrar a los estudiantes e introducir el universo microscópico |
| 1. **“¿Qué son los microorganismos y por qué son importantes?”**   Grupos pequeños de estudiantes (2 a 5 miembros) fueron dados un juego único de imagenes laminadas de diferentes ecosistemas del libro XXXX. Cada estudiante debía elegir a un microorganismo y explicarle al resto del salón su función y por qué lo eligieron. | Fomentar la realización en los estudiantes que los microorganismos juegan un rol importante en muchos ecosistemas. |
| 1. **Construye tu propio foldscope**   Cada estudiante recibió un foldscope y se le explicó al grupo cómo construir su propio microscopio paso a paso mientras yo demostraba físicamente el proceso. | Mostrarle a los estudiantes cómo construir su propio microscopio, así fomentando un sentido de apropiación, curiosidad y cuidado de su instrumento |
| 1. **Visualizar muestras preparadas**   Se le mostró a los estudiantes cómo usar el foldscope con una serie de portaobjetos con muestras fijadas que vienen incluídos en el “estuche de maestre” (rizomas, músculo, arterias, hueso, tejido foliar, pollen y bacterias) de *Foldscope Instruments*. | Enseñarle a los estudiantes como insertar y visualizar portaobjetos en su foldscope con un giro atractivo y muestras interesantes |
| 1. **Exploración**   Se invitó a les estudiantes a preparar sus propios portaobjetos de papel que vienen incluidos en el foldscope. Las muestras podían venir del material proveído por mi (flores, hojas, insectos, agua estancada, larvas de mosquitos…) o del ambiente donde el taller estaba tomando lugar. | Instruir a los estudiantes en la preparación de sus propios portaobjetos y muestras y fomentar su creatividad para seleccionar objetos del ambiente para observar al microscopio |
| 1. **La red Microcosmos**   Se presentó la red social Microcosmos a les estudiantes y se les mostró cómo hacer publicaciones y conectar con otres exploradores en todo el mundo. | Fomentar el uso del foldscope fuera del taller e incluso fuera del salón |
| 1. **Cuestionario final**   Los estudiantes contestaron un segundo cuestionario que contenía las mismas preguntas del cuestionario inicial, además de unas cuantas más sobre su experiencia en el taller y su uso del foldscope en un futuro. | Comparar respuestas entre cuestionarios iniciales y finales para evaluar el éxito del taller. De ser posible, incorporar comentarios en el próximo taller |

Supplementary 3.

**Initial Questionnaire (Spanish)**

*Institución:*

*Fecha:*

*Grupo:*

*Edad:*

*Contesta cada pregunta lo mejor que puedas.*

1. *¿Qué es un microscopio?*
2. *¿Qué son y para qué sirven los microscopios?*
3. *Alguna vez has observado algo bajo un microscopio o una lupa? Si sí, ¿qué observaste?*
4. *¿Sabes lo que es un ecosistema? Si sí, ¿por qué son importantes los ecosistemas? ¿Puedes nombrar algunos componentes del ecosistema?*
5. *En el mundo microscópico podemos observar muchos diferentes organismos que son demasiado pequeños para ver a simple vista, ¿puedes nombrar algunos?*

Supplementary 4.

Ecosystem: Understood as the variety of biological, physical and chemical processes connecting biotic: organisms, and abiotic factors: environment. From Loreau M. 2010. Linking biodiversity and ecosystems: towards a unifying ecological theory. Philosophical Transactions of the Royal Society B: Biological Sciences 365: 49–60.

Microorganism: Understood as any organism that is not visible to the naked eye due to its reduced size.

Evolution: Understood as the process through which populations accumulate change over generations (through mutation, natural selection and genetic drift).

Supplementary 5.

List of organisms and objects that participants reported wanting to observe with their foldscope after the workshop.

- Ash
- Brush
- Wax
- Coke
- Food
- Fish food
- Rotten food
- Eye cornea
- Cockroach
- Tooth
- Money
- Diseases
- Fish scales
- Sperm
- Spinach
- Spores
- Stomas
- Strawberry
- Gelatin
- Grains
- Worms
- Used dental floss
- Leaf
- Sunflower leaf
- Fungus
- Food fungus
- Ant
- Fingerprint
- Mite
- Kale
- Water
- Soapy water
- Water with yeast
- Water with dirt
- Puddle water
- Pond water
- River water
- Water from Xochimilco canals
- Dirty water
- Garlic
- Butterfly wing
- Insect wing
- Algae
- Cotton
- Animals
- Spider
- Sand
- Sugar
- Bacteria
- Biofloc
- Hair
- Dyed hair
- Cockroach head
- Coffee
- Meat
- Dandruff
- Bones
- Insect
- Eye booger
- Teardrop
- Mosquito larva
- Insect larva
- Tongue
- Wood
- Make-up
- Boogers
- mosquitoes
- Snake skin
- Nail dirt
- Orange
- Eye
- Water bear
- Bread
- Toilet paper
- Bit of paper money
- Bit of muscle
- Cat hair
- Dog hair
- Eyelash
- Flower petal
- Skin
- Onion
- Tomato
- Lice
- Dental plaque
- Plants
- Plastic
- Feather
- Polichaeta
- Pollen
- Dust
- Flea
- Hair root
- Facial hair root
- Soda
- Clothes
- Salt
- Human saliva
- Dog saliva
- Blood
- Seeds
- Chia seeds
- Sweat
- Shoe sole
- Tardigrades
- Fabric
- Earth
- Moon soil
- Pen ink
- Nail
- Cat nails
- Vegetables

Supplementary 6.

| **Supplementary Table 6.** Student answers given to the question “In the microscopic world we can observe many different organisms that are too small to observe with the naked eye. Can you name a few of these organisms?” of the initial and final questionnaires. | | | |
| --- | --- | --- | --- |
| Questionnaire | Organisms | Organism parts | Other |
| Initial questionnaire | Small animals, insects, yeast, dragonfly, bacillus, bacteria, cells, fungi, germs, microbes, microorganisms, parasites | Erythrocyte, spores, red blood cells, leaves, hair, pollen, blood | Atoms, diseases, the five kingdoms, molecules, particles, viruses |
| Final questionnaire | Bed mites, food mites, small animals, bacteria, bugs, copepods, diatoms, fleas, worms, flatworms, water mites, fungi, insects, crab larvae, mosquito larvae, larvae, yeast, microorganisms, mosquitoes, tardigrades, ostracods, parasites, lice, plankton, plants, polychaetes, pseudoscorpions, psidium, water fleas, chaetognaths, rotifers | Butterfly wing, saliva, hair, onion, sick and healthy cells, cells, erythrocytes, fish scales, sperm, fungi spores, hair follicles, fish eggs, muscles, insect parts, pollen, rhizomes | Tooth brush, food, water drop, sourdough, under nail dirt, dust, virus |
